# Genome-wide survey of HMA gene family and its characterization in wheat (*Triticum aestivum*)

**DOI:** 10.7717/peerj.14920

**Published:** 2023-03-03

**Authors:** Sadaf Zahra, Tayyaba Shaheen, Muhammad Qasim, Mahmood-ur-Rahman, Momina Hussain, Sana Zulfiqar, Kanval Shaukat, Mehboob-ur-Rahman

**Affiliations:** 1 Department of Bioinformatics and Biotechnology, Government College University, Faisalabad, Pakistan; 2 Department of Biotechnology, Faculty of Life Sciences, University of Okara, Okara, Pakistan; 3 Plant Genomics and Molecular Breeding Laboratory, National Institute for Biotechnology and Genetic Engineering College, Pakistan Institute of Engineering and Applied Sciences (NIBGE-C, PIEAS), Faisalabad, Punjab, Pakistan; 4 Department of Botany, University of Balochistan, Quetta, Pakistan

**Keywords:** Bioinformatics, Genome-wide analysis, Heavy metal toxicity, HMA gene family, Phylogenetic analysis

## Abstract

**Background:**

Abiotic stresses, particularly drought and heavy metal toxicity, have presented a significant risk to long-term agricultural output around the world. Although the heavy-metal-associated domain (HMA) gene family has been widely explored in Arabidopsis and other plants, it has not been thoroughly studied in wheat (*Triticum aestivum*). This study was proposed to investigate the HMA gene family in wheat.

**Methods:**

To analyze the phylogenetic relationships, gene structure, gene ontology, and conserved motifs, a comparative study of wheat HMA genes with the Arabidopsis genome was performed.

**Results:**

A total of 27 *T. aestivum* proteins belonging to the HMA gene family were identified in this study, with amino acid counts ranging from 262 to 1,071. HMA proteins were found to be grouped into three subgroups in a phylogenetic tree, and closely related proteins in the tree showed the same expression patterns as motifs found in distinct subgroups. Gene structural study elucidated that intron and exon arrangement differed by family.

**Conclusion:**

As a result, the current work offered important information regarding HMA family genes in the *T. aestivum* genome, which will be valuable in understanding their putative functions in other wheat species.

## Introduction

Wheat (*T. aestivum* L.), a major food crop worldwide, is cultivated on nearly 20% of agricultural land and serves as a significant source of food for 30% of the world’s population (Vasil, 2007). The global wheat output (growth and yield) is adversely influenced by environmental stresses such as water scarcity and toxic metals, *etc*. (Javed et al., 2016). Plants have evolved a variety of adaptation strategies, to protect themselves from harsh environmental conditions (Raza et al., 2019). Plant biologists have long been fascinated by the regulation and expression of many genes for improved crop resilience to biotic and abiotic stresses, along with increased productivity. Drought and heavy metal toxicity are among the abiotic stresses that have posed a severe threat to crop yield globally (Bari et al., 2018). Drought is one of the most common stresses in heavy metal contaminated environments (Barceló & Poschenrieder, 2004) and causes a variety of biochemical and physiological changes in plants (Zahra et al., 2021a). Heavy metal accumulation and transportation to grain has a detrimental effect on human health as well. Therefore, it is pivotal to understand the mechanism of metal accumulation and transport in the grain to mitigate this phenomenon (Ahmad et al., 2018).

Plants selectively sense environmental stimuli and resultantly activate signaling cascades to assemble an overall response for their survival, which is mediated by complex signaling networks (Cappetta et al., 2020). Heavy metal associated (HMA) protein, familiar as P_1B_-ATPase, participates in absorbing and transporting heavy metal ions (Cu^2+^, Co^2+^, Zn^2+^, Pb^2+^ and Cd^2+^) by combining ATP hydrolysis with metal ion transport across membranes (Imran et al., 2016; Zhang et al., 2021). Currently, the number of identified *HMA* genes are eight in *A. thaliana*, nine in rice *(Oryza sativa* L.), 11 in sorghum (*Sorghum bicolor* L.), 11 in maize (*Zea mays* L.), 20 in soybean (*Glycine max* L.), 17 in *Populus trichocarpa and* 21 in barley (*Hordeum vulgare* L.) (Zhang et al., 2021). *HMA* domain genes are vital for the spatiotemporal transit of metal ions that bind to several enzymes and cofactors throughout the cell (He et al., 2020). It is worth noting that HMA affects not only heavy metal transport but also plant growth and development (Grispen et al., 2011).

Wheat is sensitive to heavy metals. Heavy metals trigger different responses in wheat, leading to yield losses in wheat (Rizvi et al., 2020). However, data regarding this gene family in hexaploid wheat is scanty. Limited similarities have been found in the mechanisms of both drought and heavy metal tolerance strategies in plants (Islam & Sandhi, 2022). Signaling pathways activate proteins that make transporters, proteases, ROS detoxifying enzymes (alternative oxidase, glutathione peroxidase, glutathione reductase, copper-zinc superoxide dismutase, glutathione S transferase and chaperones (Zhang & Sonnewald, 2017)), which help plants to ameliorate stress. Under abiotic and biotic stress, the molecular processes and signal transduction pathways of the *HMA* family of genes, their function in shielding plants from pathogens and environmental stresses are currently poorly known. However, abiotic stress is intimately linked to the HMA gene family (He et al., 2020). In a previous study, the expression of the yeast AcHMA1 gene improved yeast cell’s resilience to stresses such as drought, alkali, salt, and oxygen (Sun et al., 2014).

Several *HMA* genes were found to play different roles in various species of plants, as *OsHMA2* is linked to zinc loading in vascular tissue and tonoplast localization in rice (Yamaji et al., 2013). *OsHMA3*, which is found in tonoplasts, transports Cd to the roots, whereas *OsHMA4* transports copper (Huang et al., 2016; Zhang et al., 2021). HvHMA1 aids in the transfer of zinc and cadmium into barley grain (Mikkelsen et al., 2012). There are evidences that *HMAs* play a vital role in heavy metal transmembrane trafficking. However, little is known about *HMAs* in wheat. This study reports a complete identification of *HMA* genes in wheat including syntenic examination, gene structures analysis, and conserved motif analysis. This study may lay the foundation for further investigate the putative functions of the HMA gene family in wheat.

## Materials and Methods

### Retrieval of protein sequences

HMA protein sequences of Arabidopsis (Table 1) and wheat (Table 2) were retrieved from the NCBI database (http://www.ncbi.nlm.nih.gov/). These sequences were further verified from The Arabidopsis Information Resources (TAIR) (http://www.arabidopsis.org/index.jsp) while the Phytozome database of wheat (*T. aestivum*) was used to confirm these proteins in wheat using online server (https://phytozome.jgi.doe.gov/).

**Table 1:** Physio-chemical property of heavy metal associated (HMA) proteins in Arabidopsis.

S. No.	Gene name	Transcript name	Gene ID	Translation length (a.a)	Protein accession no.	Chr.	Subcellular location	Strand
1.	HMA1	AT4G37270.1	19648322	819	NP_195444.1	4	Plasma membrane	Reverse
2.	HMA2	AT4G30110.1	19643720	951	NP_001320088.1	4	Plasma membrane	Reverse
3.	HMA3	AT4G30120.1	19645860	542	NP_001328455.1	4	Plasma membrane	Reverse
4.	HMA4	AT2G19110.1	19641614	1,172	NP_179501.1	2	Plasma membrane	Forward
5.	HMA5	AT1G63440.1	19650287	995	NP_176533.1	1	Chloroplast	Forward
6.	HMA6.1	AT4G33520.2	19645604	949	NP_974675.1	4	Chloroplast	Forward
7.	HMA6.2	AT4G33520.3	19645605	949	NP_974676.1	4	Plasma membrane	Forward
8.	HMA7	AT5G44790.1	19665355	1,001	NP_199292.1	5	Chloroplast	Reverse
9.	HMA8	AT5G21930.1	19665627	883	NP_001237371.2	5	Chloroplast	Forward
10.	HMA8.2	AT5G21930.2	19665628	883	NP_001031920.1	5	Plasma membrane	Forward

**Table 2:** Physio-chemical properties of heavy metal associated (HMA) proteins in wheat.

S. No.	Gene name	Transcript name	Gene ID	Translation length (a.a)	Protein accession no.	Chr.	Subcellular location	Strand
1.	HMA1	Traes_2AL_D0EABF355.2	31873047	863	KAF7003072.1	2A	Plasma membrane	Forward
2.	HMA2	Traes_2BL_19B3E60AA.1	31802866	845	KAF7010487.1	2B	Plasma membrane	Forward
3.	HMA3	Traes_2DL_51FF05F66.1	32015465	888	KAF7017839.1	2D	Plasma membrane	Reverse
4.	HMA4	Traes_4AS_622EEFE10.2	31758612	648	KAF7041053.1	4A	Chloroplast	Forward
5.	HMA5	Traes_4BL_89775421A.2	31775420	262	KAF7050535.1	4B	Extracellular	Forward
6.	HMA6	Traes_4DL_385639507.1	31802013	604	KAF7029642.1	4D	Chloroplast	Reverse
7.	HMA7	Traes_5AL_C89EEBE50.2	31899687	558	XP_044438068.1	5A	Chloroplast	Reverse
8.	HMA8	Traes_5BL_D6C3DC326.1	31786522	829	XP_044401945.1	5B	Plasma membrane	Reverse
9.	HMA9	Traes_5BL_F83C809F0.1	31807288	458	XP_044424302.1	5B	Cytoplasmic	Forward
10.	HMA10	Traes_5DL_91C1891D3.1	31858473	632	KAF7074611.1	5D	Plasma membrane	Forward
11.	HMA11	Traes_6AS_6F306F27E.1	31768608	974	KAF7089256.1	6A	Cytoplasmic	Forward
12.	HMA12	Traes_6AS_9321C1C5B.2	31961570	1,074	KAF7084039.1	6A	Plasma membrane	Reverse
13.	HMA13	Traes_6BS_9A12C2A1D.1	31766484	837	KAF7098168.1	6B	Plasma membrane	Froward
14.	HMA14	Traes_6BS_A8B960E60.1	31836000	813	XP_044412145.1	6B	Cytoplasmic	Reverse
15.	HMA15	Traes_6DS_26C5A0A44.1	31904930	980	KAF7084123.1	6D	Plasma membrane	Forward
16.	HMA16	Traes_6DS_9FA053DF8.2	31748333	862	KAF7098167.1	6D	Plasma membrane	Reverse
17.	HMA17	Traes_7AL_7A2639A1B.2	31943896	916	XP_044424773.1	7A	Plasma membrane	Forward
18.	HMA18	Traes_7AL_8304348B7.1	32021814	790	KAF7097469.1	7A	Extracellular	Forward
19.	HMA19	Traes_7DL_A5269C73F.2	31916999	1,061	KAF110499.1	7D	Plasma membrane	Reverse
20.	HMA20	Traes_7BS_8EC4B41E4.1	31834193	737	KAF7100926.1	7B	Chloroplast	Forward
21.	HMA21	Traes_7DS_04F16455B.1	31749939	804	XP_044426682.1	7D	Plasma membrane	Reverse
22.	HMA22	Traes_7AS_766146E70.1	31803725	806	KAF7095199.1	7A	Plasma membrane	Reverse
23.	HMA23	Traes_7BL_EFF0E2E31.1	31766590	718	XP_037446570.1	7B	Chloroplast	Reverse
24.	HMA24	Traes_7DL_DF97DD324.1	31923629	959	XP_020190440.1	7D	Chloroplast	Forward
25.	HMA25	Traes_7BL_041308E74.3	31832822	636	XP_037464768.1	7B	Plasma membrane	Reverse
26.	HMA26	Traes_7AL_84D5BAE85.1	31955716	638	XP_037460689.1	7A	Plasma membrane	Forward
27.	HMA27	Traes_7DL_271C7BED5.1	31915788	500	XP_020187387.1	7D	Chloroplast	Forward

Protein BLAST (Blastp) tool of NCBI was used to find similar sequences in wheat, using 50% identity as a threshold. Further, the motif finder online tool (https://www.genome.jp/tools/motif/) was used to confirm that these genes contain HMA domains. Peptide sequences not possessing HMA domains were deleted.

### Determination of HMA protein properties

Different protein properties such as peptide length (a.a), DNA strand, chromosomal location, transcript ID, and subcellular locations were described in wheat while using Arabidopsis as model genome using online tools Expasy (https://web.expasy.org/protparam/) and plant Ensemble tool (https://plants.ensembl.org/).

### Sequence alignment and construction of phylogenetic tree

Full-length sequences of HMA proteins of Arabidopsis and wheat were aligned using ClustalX (Thompson et al., 1997) and were used for the construction of phylogenetic tree according to the neighbor-joining method of Saitou & Nei (1987) at 1,000 bootstrap value using the MEGA7 tool (Katsu et al., 2021).

### Gene structure analysis

To observe the pattern of exon and intron organization in the HMA gene family, an online tool, gene structure display server GSDS 2.0 (http://gsds.cbi.pku.edu.cn/) was used. CDS and genomic sequences of wheat and Arabidopsis were used as input files however the default parameters of the tool remained unchanged.

### Conserved motif analysis

Conserved motifs and HMA proteins were analyzed using the online tool MEME SUITE version 4.8.2 (https://meme-suite.org/meme/doc/release-notes.html) according to the method described by Li & Dewey (2011). These motifs were illustrated in the corresponding branch of the phylogenetic tree. Default parameters set were, a maximum number of motifs = 10, minimum motif width = 6 and maximum motif width = 50, minimum sites per motif = 2, and maximum sites per motif = 37.

### Prediction of subcellular location

Subcellular locations of HMA proteins in wheat were determined using the tool WoLF PSORT (https://wolfpsort.hgc.jp/). An excel sheet was prepared to present the information about gene names and their location and their 14 nearest neighbors using the WoLF PSORT data and then TBTool (Guo et al., 2007) was used to create the heatmap.

### Synteny analysis

An online tool of synteny viewer tool (tools.bat.infspire.org/circoletto/) was used to find the evolutionary relationship between Wheat and Arabidopsis HMA proteins. Protein sequences of all the HMA downloaded proteins were used as input files to compare the wheat genome with Arabidopsis using default parameters (Darzentas, 2010).

### Identification of homologous pairs and calculating Ks/Ka values

Homologous pairs of *HMA* genes were manually selected from the phylogenetic tree and Ks/Ka values were calculated using TBTool using genomic sequences, protein sequences, and gene duplication pairs as input files.

## Results

Sequencing of the wheat genome has made it possible to identify the *HMA* genes in this important cereal crop. The *HMA* gene family was not previously characterized in wheat. Therefore, we selected *HMA* gene family and performed genome wide survey in wheat (*T. aestivum*). We used Arabidopsis HMA proteins using the blastp tool to find similar sequences in wheat. A total of 27 genes of the *HMA* gene family were found in wheat in this study.

### Characteristics of Arabidopsis HMA proteins

In Arabidopsis *HMA* gene family was comprised of 10 members. In Arabidopsis *HMA* genes were located on all the chromosomes except chromosome 3. Amino acid length of HMA proteins was ranged from 542 to 1,172 (Table 1). The subcellular location analysis indicated that six HMA proteins were present in the plasma membrane and four in the chloroplast. Four genes were located on reverse strand and six on the forward strand.

We identified 27 HMA proteins in wheat. Gene location indicated that wheat *HMA* genes were present on the 4^th^, 5^th^, 6^th,^ and 7^th^ chromosomes. Out of 27 proteins, 15 were present in the plasma membrane, 10 in the chloroplast, and two in extracellular locations. Seventeen proteins were present on the forward strand and 10 on the reverse strand. Amino acid length of wheat HMA proteins ranged from 262 to 1,071 (Table 2).

### Sequence alignment and phylogenetic association

**Figure 1:**
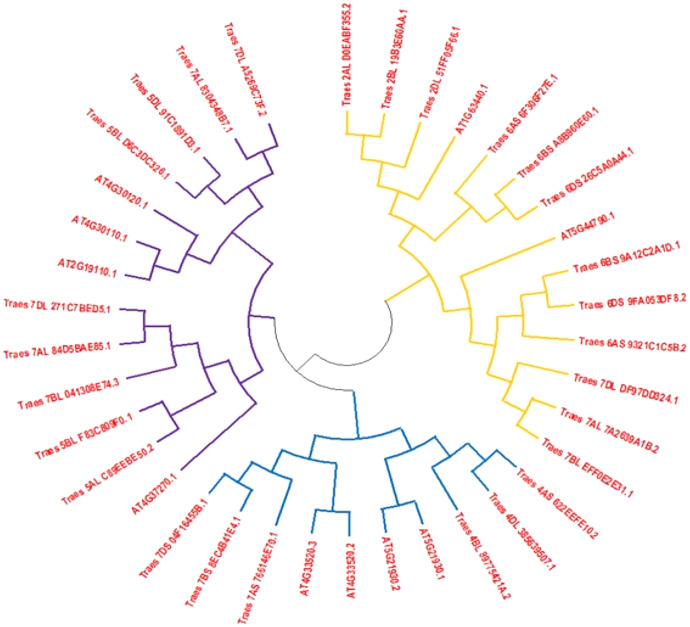
An unrooted phylogenetic tree of Arabidopsis (At) and wheat (Ta). Tree of HMA proteins constructed by the following of neighbor-joining method with MEGA6.0 software. Three subclasses were differentiated by orange, blue and dark blue colors.

Full-length HMA protein sequences from wheat and Arabidopsis obtained from different databases were used to construct the phylogenetic tree to assess the phylogenetic association among both plant species. The phylogenetic tree indicated that HMA proteins were distributed in three subgroups. Clad one was the largest subgroup containing 14 proteins that were belonging to both species, second clad consisted of 11 members and third clad was comprised of 13 members (Fig. 1).

### Gene structure analysis

**Figure 2:**
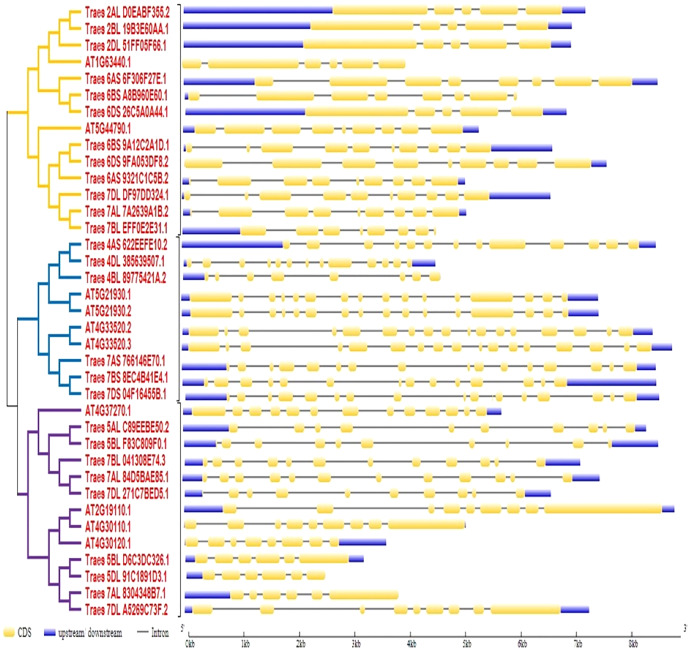
Gene structure analysis of HMA genes in wheat and Arabidopsis. Exon/intron pattern was predicted by gene structure display server 2. CDS/Exons were presented with yellow color, intron with the black line, and upstream/downstream with blue color.

To predict the exon-intron organization in wheat and Arabidopsis *HMA* genes, CDS and genomic DNA were used as input files. The organization pattern of intron/exons in *HMA* genes was displayed to the relative branch in the phylogenetic tree. It was observed that several introns and exons varied among these genes. Arabidopsis genes *ATG33520.2* and *ATG33520.3* showed the largest number of exons (17) whereas four wheat genes showed the fewest (five) exons. Further, it was observed that closely related members in a subclass showed similar intron-exon pattern (Fig. 2).

### Conserved motif analysis

**Figure 3:**
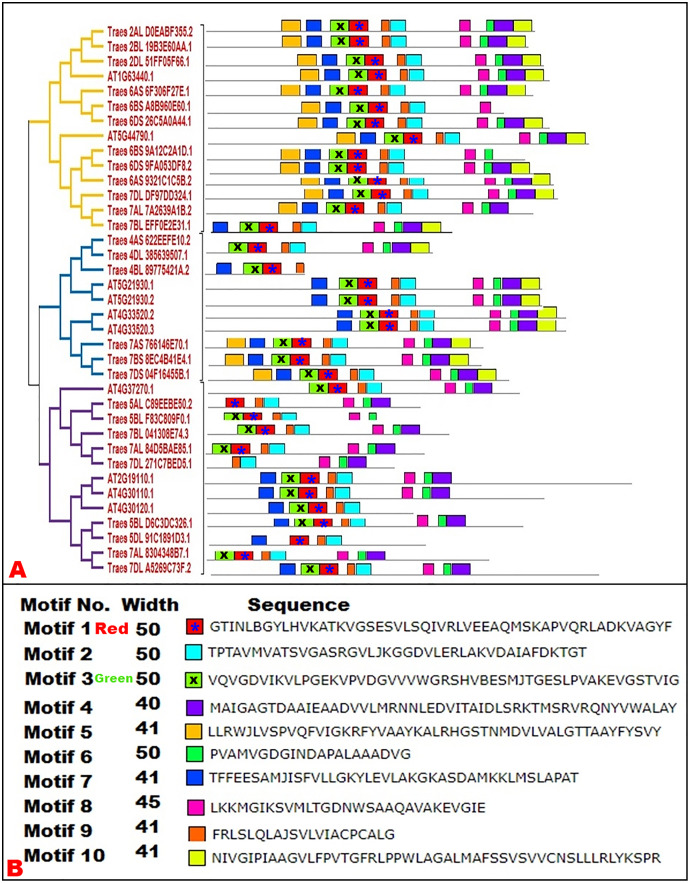
Conserved motifs of HMA proteins in wheat and Arabidopsis. Each motif was distinct from the other and represented by various colors, discovered by MEME Suit tool. (A) An asterisk (*) in the red and ‘x’ in the green color box is to distinguish between the colors for greater accessibility.

To predict the conserved domains in wheat and Arabidopsis HMA proteins, motif analysis was performed. Ten distinct motifs were discovered in both plant species. We selected the motif width from 10 to 50 as default parameters however it was noted that motif width was ranging from 40 to 50 indicating that highly conserved regions in HMA proteins were present. Each motif was displayed to the concerned protein on the phylogenetic tree to explore the motif pattern according to the phylogenetic association. It was noted that closely linked proteins in phylogenetic tree were showing the same expression pattern as the motifs falling in different subgroups of tree (Fig. 3). Our results regarding the conservation of motifs within subgroups were supported by previous studies on different gene families (Azeem et al., 2018; Waqas et al., 2019).

### Prediction of subcellular locations

**Figure 4:**
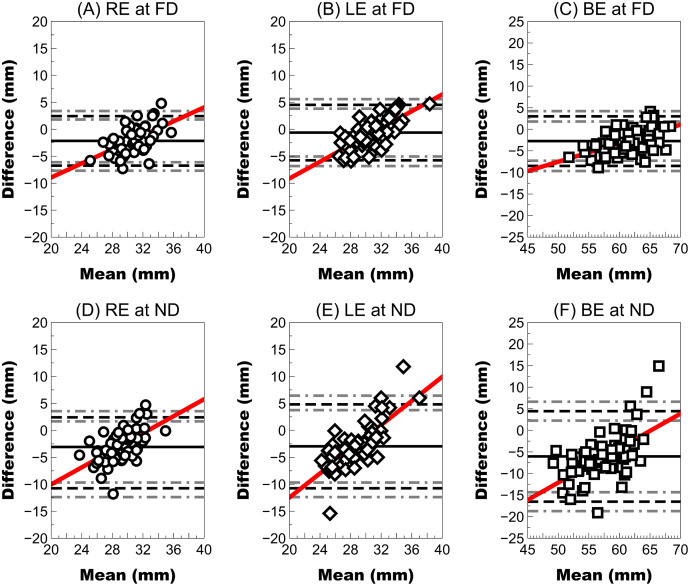
Subcellular location of HMA proteins in Arabidopsis and wheat. Heatmap was constructed by using TBTool. Where NUC, nucleus; Plas, plasma membrane; Cyto, cytoplasm; VAC, vacuole; ER, endoplasmic reticulum; CHL, chloroplast; GOLG, golgi body; Mito, mitochondria; EXTRA, extracellular.

Subcellular locations of 27 HMA proteins were predicted in various subcellular components such as nucleus, plasma membrane, cytoplasm, vacuole, endoplasmic reticulum, chloroplast, golgi bodies, mitochondria, and extracellular locations. Results indicated that most of the proteins were present in plasma membranes followed by endoplasmic reticulum and vacuoles whereas lowest proteins were located on extracellular locations and golgi bodies (Fig. 4).

### Synteny analysis

**Figure 5:**
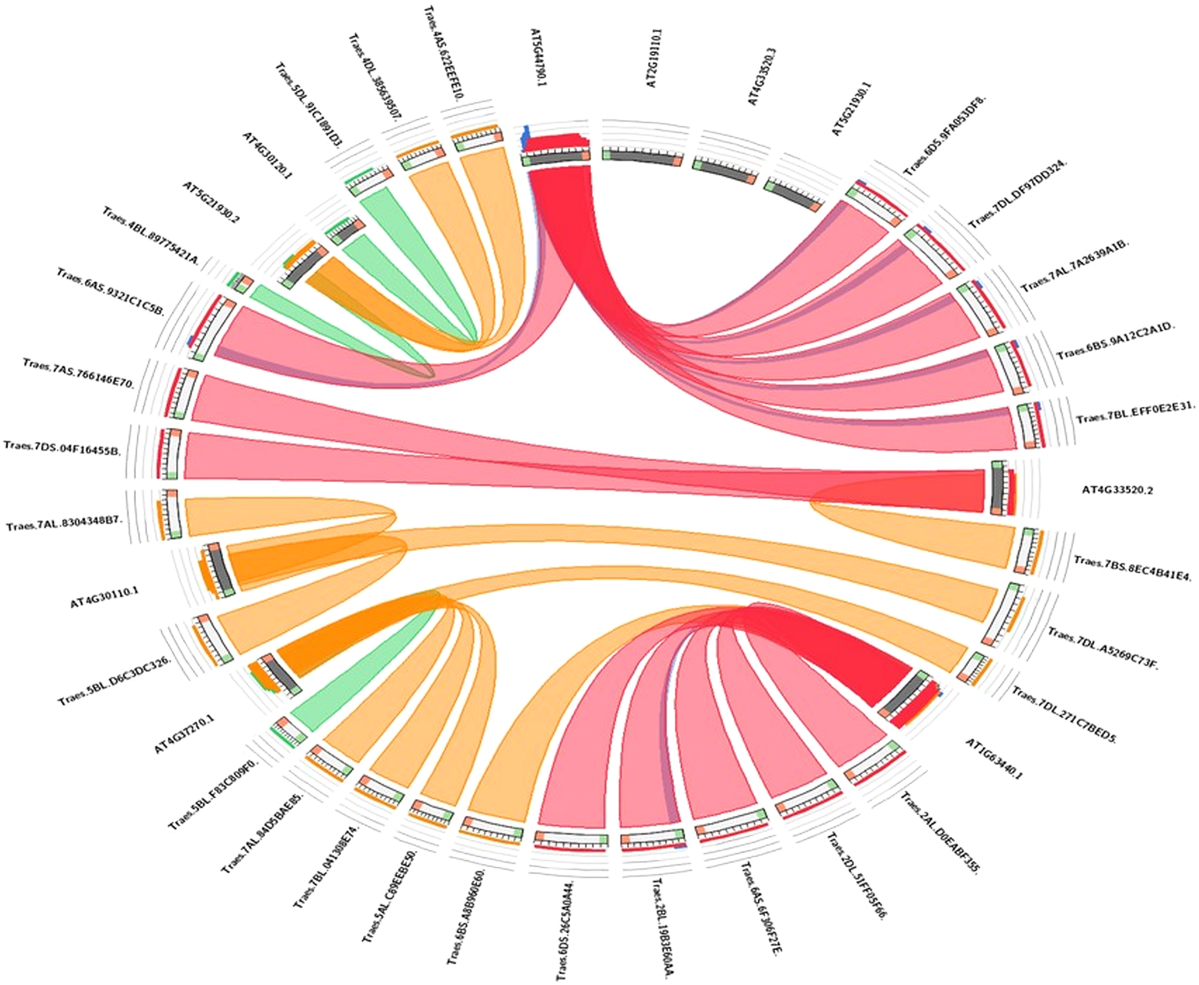
Visualization of the sequence similarity of wheat *HMA* genes with Arabidopsis *HMA* genes. In the circuletto tool wheat HMA proteins were used as query and Arabidopsis proteins as comparative files as per default parameters of the tool. Colored lines which connect two regions indicate syntenic regions between Arabidopsis and wheat.

The evolutionary link of Arabidopsis *HMA* genes with wheat genes was assessed through a micro-syntenic tool. It was concluded that most of the wheat *HMA* genes and Arabidopsis *HMA* genes have similar evolutionary origin (Fig. 5). *Traes.7AS.766146E70.1* and *Traes.7DS.04F16455B.1* were originated from Arabidopsis gene *AT4G33520.2*. Similarly, Arabidopsis gene *AT4G30110.1* gave rise to wheat *Traes_5BL_D6C3DC326.1, Traes_7AL_8304348B7.1* and *Traes_7BS_8EC4B41E4.1. Traes_6DS_9FA053DF8.2, Traes_7DL_DF97DD324.1, Traes_7AL_7A2639A1B.2, Traes_6BS_9A12C2A1D.1* and *Traes_7BL_EFF0E2E31.1* were evolutionary originated from *AT2G19110.1*.

### Gene ontology

**Figure 6:**
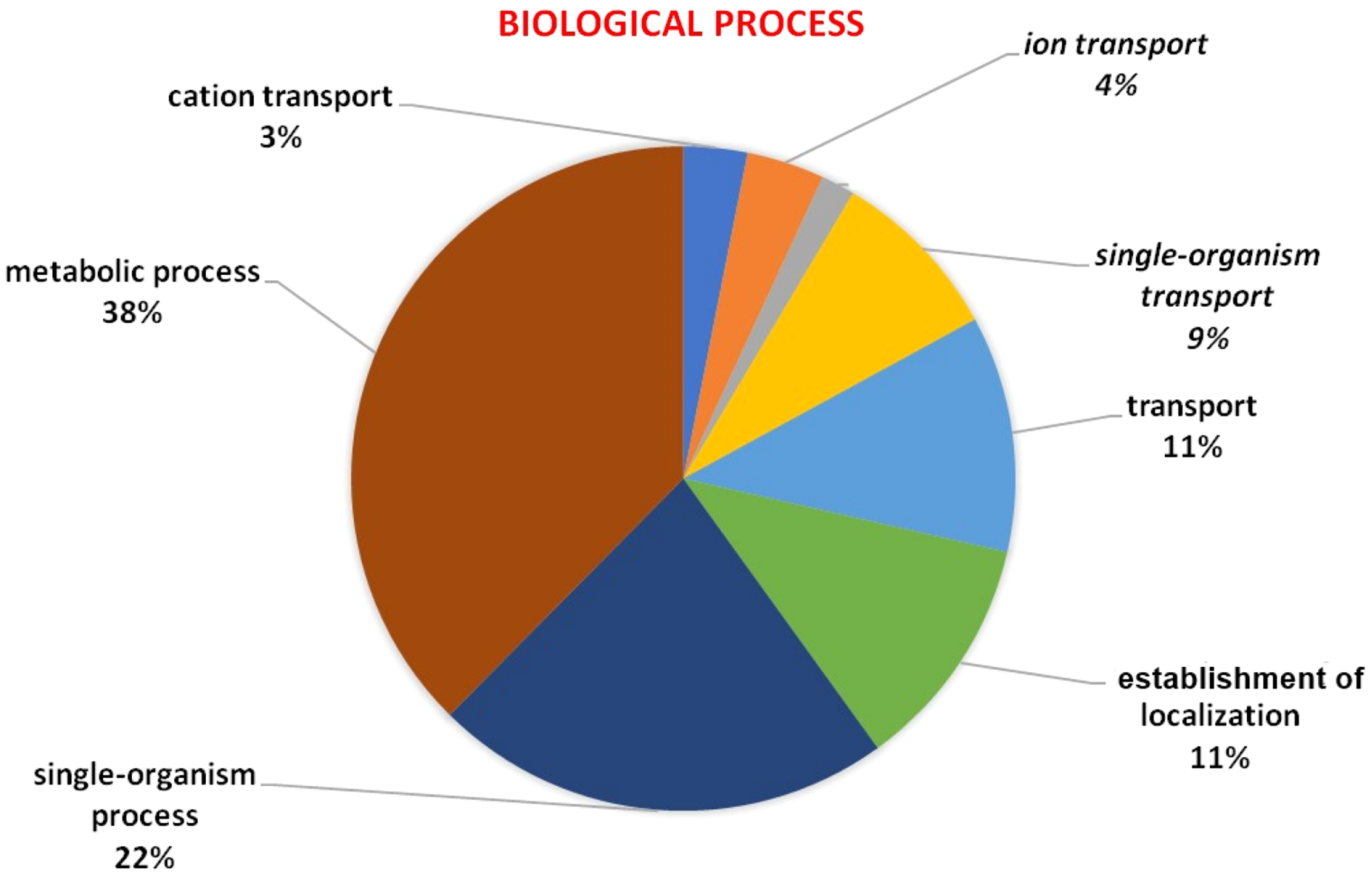
GO of biological process determined through Blast2GO tool using the Arabidopsis, and wheat HMA proteins as a query. Various biological processes carried out by these genes were distinguished by different colors.

**Figure 7:**
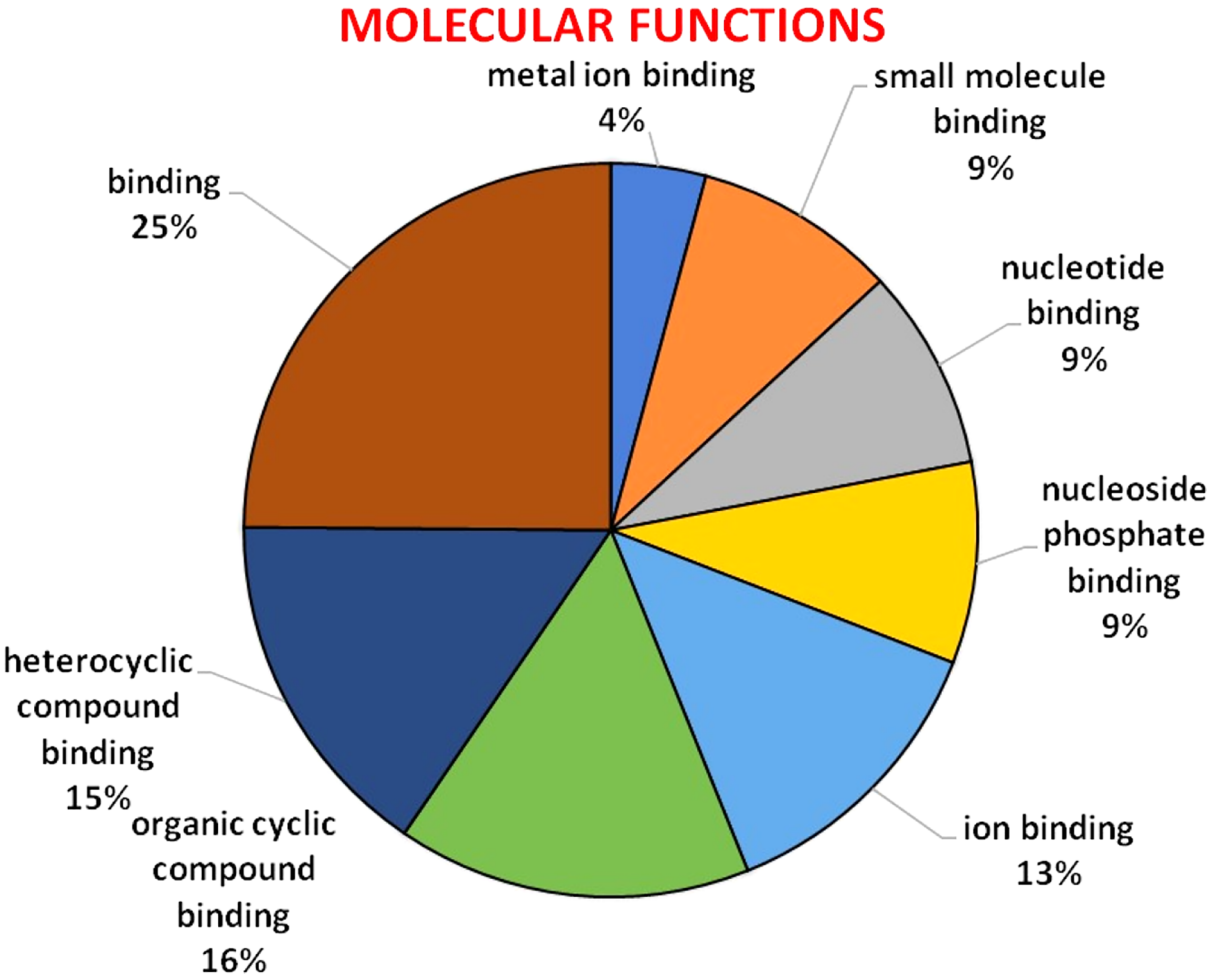
GO of molecular functions determined through Blast2GO tool using the Arabidopsis, and wheat HMA proteins as a query. The genes were distinguished by different colors.

GO analysis was used to describe the functions of a gene such as involvement in biological processes, molecular activities of the gene products, and location of these activities. GO analysis indicated that *HMA* genes were mainly involved in metabolic processes, single-organism process, localization establishment, single organism transport, metal ion, ion, and cation transport (Fig. 6). Molecular functions of *HMA* genes observed through GO tools indicated that these genes are mainly involved in various types of binding activities. The percentage of binding with different compounds is shown in Fig. 7. *HMA* genes mainly bind with organic cyclic compounds, heterocyclic compounds, ion binding, nucleotides, and nucleoside bindings.

Ka/Ks ratio determines the ratio of beneficial mutations and neutral mutations present on a set of homologous genes. This ratio also indicates the net balance between beneficial and deleterious mutations. Six gene pairs were duplicated in wheat belonging to the *HMA* family. Ka, Ks, and Ka/Ks ratio was calculated using TBtool. The ratio of Ka/Ks value in the Table 3 indicated that all the six homologous pairs showed a value less than one. Ka/Ks greater than one expressed positive selection, less than one indicates purified/stable selection and equal to one indicates neutral selection. Hence according to results, Ka/Ks value of all the pairs is below one which means HMA genes have the stable and purifying selection.

**Table 3:** Estimated time of divergence of wheat heavy metal associated (HMA) genes.

S. No.	Seq_1	Seq_2	Ka	Ks	Ka/Ks	T (MYA)
1.	Traes_7AL_7A2639A1B.2	Traes_7BL_EFF0E2E31.1	0.007469	0.118871	0.062836074	9.90589E−09
2.	Traes_7BS_8EC4B41E4.1	Traes_7DS_04F16455B.1	0.008408	0.042414	0.1982277	3.53451E−09
3.	Traes_5AL_C89EEBE50.2	Traes_5BL_F83C809F0.1	0.033487	0.10141	0.330218025	8.4508E−09
4.	Traes_7AL_84D5BAE85.1	Traes_7DL_271C7BED5.1	0.001769	0.050569	0.03498181	4.21409E−09
5.	Traes_5BL_D6C3DC326.1	Traes_5DL_91C1891D3.1	0.181692	0.332826	0.545906617	2.77355E−08
6.	Traes_7AL_8304348B7.1	Traes_7DL_A5269C73F.2	0.007949	0.090577	0.087758967	7.54811E−09

## Discussion

Abiotic stresses like drought and heavy metal toxicity have presented a severe challenge to global food production. The impacts of heavy metals on plants and their ability to withstand metal toxicity have been widely studied (Gao et al., 2020). The consequences of heavy metal stress on a plant’s ability to deal with other environmental difficulties, including water scarcity, have received less attention (Barceló & Poschenrieder, 2004). Previously, a mutation in HMA domain in the chimeric allele of the drought resistant wheat mutant NN1-M-700 was responsible for drought stress tolerance (Zahra et al., 2021b). Heavy-metal-associated domain (HMAD) has been found to have a variety of vital roles in Arabidopsis, and significant progress was achieved in identifying *HMA* genes in many other plants (Li et al., 2016; Sutkovic et al., 2016; Wu et al., 2019; Zhang et al., 2021). However, reports regarding this gene in hexaploid wheat are meager. In wheat and other crop species, the *HMA* gene family plays a significant role in heavy metal transport and abiotic stress tolerance (Wu et al., 2019).

Drought is known as one of the most prevalent strain in metal-contaminated ecosystems (Sitko et al., 2021). Various plant indicators are used to compare heavy metal exposure with drought stress, such as photosynthetic performance and stomatal behavior, photosynthetic pigment, proline, and peroxidase. Abiotic stress crosstalk includes the ROS (reactive oxygen species) signal and the antioxidant system, as well as drought stress and heavy metal stress (Khan, Ahmed & Shah, 2022). Metal-rich soils often have poor structure, reduced bacterial activity, and minimal organic matter content, resulting in inadequate moisture-holding capacity (Derome & Nieminen, 1998; Wang et al., 2007).Toxic metal exposure has also been shown to affect plant traits essential for plant-water associations, including abscisic acid (ABA) concentrations (Barceló & Poschenrieder, 1986), cell wall elasticity (Barceló & Poschenrieder, 1986), root elongation (Kahle, 1993), organic matter allocation to roots (Ryser & Emerson, 2007), and root permeability for water (Ryser & Emerson, 2007; Przedpelska-Wasowicz & Wierzbicka, 2011). Furthermore, in metal-exposed plants, hydraulic and stomatal conductance has been reported to be reduced (Lamoreaux & Chaney, 1977; Disante, Fuentes & Cortina, 2011). Similar findings were observed in EMS mutant lines of NN-1 wheat (NN1-M-363, NN1-M-506, NN1-M-700, NN1-M-701, and NN1-M-1621) (Zahra et al., 2021b). All of the above physiological processes may reduce water uptake in metal-stressed plants, aggravating the consequences of drought stress.

In the present research, we provided a complete overview of HMA gene family in wheat. Further, we analyzed the phylogenetic relationship, subcellular location, gene structure, conserved motifs, identification of homologous pairs, and Ka/Ks ratio under drought conditions. A plant’s sensitivity to various stresses cannot always be inferred from their responses to specific stresses (Mittler, 2006). Despite substantial study of the effects of drought and metals on plants as separate stimuli, experiments subjecting plants to both stresses at the same time are rare. For annual plants like wheat and rye (Klimov, 1985), sunflower (Krizek, Foy & Wergin, 1988), and barley (Krizek, Foy & Wergin, 1988), metal stress and drought stress have been demonstrated to have synergistic growth-reducing effects (Krizek, Foy & Wergin, 1988). In this study, the phylogenetic tree domenstrated six homologous pairs of HMA genes in the wheat genome. Similar findings were published in another study on wheat by (Zhou et al., 2019). According to gene ontology, the activity or action done by a gene product is determined by its molecular function. In general terms, a molecular function, is a process carried out by a single molecular mechanism through direct physical contact with other molecular entities.

Furthermore, the distribution pattern of intron and exon is a significant tool to study comparative genomics in order to acquire understanding about a gene family, because it supports the evolutionary link of a gene with its predecessors (Waqas et al., 2019). It was observed that several introns and exons varied among these genes. Arabidopsis genes *ATG33520.2* and *ATG33520.3* showed the largest number of exons (17) whereas four wheat genes showed the fewest (5) exons. Further, it was observed that closely related members in a subclass showed similar intron- exon pattern (Zhang et al., 2019). To check the significance of HMA proteins in plant growth and development, we examined their distribution in several subcellular components. Locations of twenty-seven identified HMA proteins were predicted in various subcellular components such as the nucleus, plasma membrane, cytoplasm, vacuole, endoplasmic reticulum, chloroplast, golgi bodies, mitochondria, and extracellular locations. These proteins were shown to be abundant in the plasma membrane, demonstrating their importance in metal ion transport. Our findings are comparable with those of Zhou et al. (2019), who showed similar results in wheat HMA proteins. Variances in gene structure among members of the same class may be due to differences in evolutionary history, and these proteins may have novel functional properties (Yang et al., 2019).

The current findings show that HMA proteins have a wide range of activities. It has also been shown that there is a phylogenetic specific pattern of conserved domains (Azeem et al., 2018; Waqas et al., 2019). This pattern of conserved motifs suggested that HMA genes shared a recent common ancestor. Furthermore, the occurrence of conserved motifs leads to functional conservation and gene duplication processes in plants (Waqas et al., 2019). In polyploids, gene and genome duplication is a dominant factor in the evolution of complexity and diversity. Conserved motifs also indicate the variety of domain design, which has been used to retain domains outside the key parts of HMA genes, and play a vital role in protein function (Du et al., 2013; Tan et al., 2020). Various HMA proteins including the *A. thaliana* AtHMA1 protein, were shown to be involved in zinc/cadmium transport and chloroplast copper mobilization (Moreno et al., 2008). Furthermore, HvHMA in barley grains (Mikkelsen et al., 2012), studies on expression of *OsHMA1* in rice (Zhou et al., 2019), and analysis of Arabidopsis *HMA2* gene (Eren & Arguello, 2004) shown their role in important cellular processes. In wheat, *TaHMA2* is restricted to the plasma membrane and promotes Zn and Cd translocation from the root to the shoot (Tan et al., 2017).

Proteins from Arabidopsis (AtHMA3), rice (OsHMA3), and wheat (TaHMA3) are found in tonoplasts and are involved in transport of Zn and Cu to the vacuole (Zhang et al., 2021). In *Brassica juncea*, the HMA4 gene promotes heavy metal transport and binding, as well as increasing heavy metal resistance in yeast and *E. coli* (Wang et al., 2019). AtHMA5 mediates Cu transport from roots to the leaves or root detoxification (Zhou et al., 2019). Cu is transported to chloroplast envelope AtHMA6 (also known as PAA1), whereas it is transported into the thylakoid lumen to provide plastocyanin by AtHMA8 (PAA2) (Zhang et al., 2019). Cu is transported to ethylene receptors and Cu homeostasis in seedlings are mainly mediated by AtHMA7 (Binder, Rodríguez & Bleecker, 2010). Furthermore, studies with numerous species, including *A. thaliana* (Eren & Arguello, 2004), *O. sativa* (Huang et al., 2016), *Noccaea caerulescens* (Lochlainn et al., 2011), *Sedum alfredii* (Zhang et al., 2016), and *Sedum plumbi zincicola* (Liu et al., 2017) have reports on proteins like HMA2, HMA3, HMA4, HMA5 or HMA9.

Prior investigations have demonstrated that several similar proteins are engaged in the transport of different heavy metals and are responsible for the cross-tolerance process when combined with antioxidative enzymes. They assist plants in adapting to a wide range of stresses (Zschiesche et al., 2015; Cowan et al., 2018; Zhang et al., 2020). In *Quercus suber and Coriandrum sativum* L, however, the presence of large amounts of Zn and Cd reduced the impact of water stress on photosynthesis, stomatal conductance, and relative water content (Khan et al., 2021; Disante, Fuentes & Cortina, 2011). Metal contamination of the substrate decreased the effect of substrate moisture on white birch growth when the water supply was adequate (Santala & Ryser, 2009). The Ka/Ks ratio, also known as the dN/dS ratio, is the ratio of the number of nonsynonymous substitutions per nonsynonymous site (Ka) in a certain time period to the number of synonymous substitutions per synonymous site (Ks) in the same period. According to the current findings, the synonymous/nonsynonymous ratio was greater than one in all of the chosen homologous pairs, indicating that selection among *HMA* genes in wheat is stable and purified. However, because no data on *HMA* genes in wheat was previously available, the results were not compared.

## Conclusions

In the current study, the comprehensive identification of HMA genes in wheat (*T. aestivum* L) along with their syntenic analysis, gene structure, conserved motifs analysis, and Ka/Ks values were investigated. The result revealed a total of 27 wheat proteins belonging to the HMA gene family, ranging in amino acid count from 262 to 1,071. The study examined the specific functions of the HMAD gene family in drought-stressed wheat. The phylogenetic tree revealed that HMA proteins were divided into three subgroups, with closely related proteins in the tree displaying the same expression pattern as motifs from different subgroups. Gene structural analysis revealed that intron and exon arrangement was family-specific. Our results offer a base for further investigation on the crosstalk of molecular mechanisms of HMA genes under abiotic stress and heavy metal conditions. In future, this research might be used to better describe the significance of the HMA gene family in wheat and other crops by manipulating stress responsive genes.

## Supplemental Information

10.7717/peerj.14920/supp-1 Supplemental Information 1 Wheat genomic sequences.

10.7717/peerj.14920/supp-2 Supplemental Information 2 Wheat HMA coding sequences.

10.7717/peerj.14920/supp-3 Supplemental Information 3 Wheat and Arabidopsis HMA protein sequences.

## Additional Information and Declarations

### Data Availability

The following information was supplied regarding data availability: The raw data is available in the Supplemental Files.
